# 2.K. Round table: Navigational health literacy. Perspectives from Austria, Germany and Switzerland

**DOI:** 10.1093/eurpub/ckac129.098

**Published:** 2022-10-25

**Authors:** Organised by: Bielefeld University, School of Public Health, EUPHA Working Group on Health Literacy, Chair persons: Jürgen Pelikan (Austria)

## Abstract

**Background:**

Theoretical and empirical studies indicate that a lack of transparency and highly complexity makes it difficult for patients to navigate healthcare systems. This requires the competence to access, understand, appraise, and apply information about the healthcare system, its organizations and proceedings, i.e., navigational health literacy. Since little is known about navigational health literacy, partly due to a lack of measurement tools, a new instrument measuring navigational health literacy was developed as part of the Health Literacy Population Survey Project 2019-2021 (HLS19). It was applied in national health literacy surveys of the three German-speaking countries Austria, Germany and Switzerland among others. The empirical findings obtained in the three German-speaking countries and further perspectives on the topic will be presented and discussed in the workshop.

**Objectives of the workshop:**

The workshop on navigational health literacy has three key objectives: 1) It gives an overview on the newly developed instrument for measuring navigational health literacy in HLS19, its development and validation; 2) it aims to discuss the distribution of navigational health literacy among the Austrian, German, and Swiss population and to highlight barriers and challenges for patients regarding navigation health literacy; and finally 3) it proposes steps to promote and strengthen navigational health literacy.

**Added value of the workshop:**

The workshop is designed to enhance the understanding of navigational health literacy and to propose future scenarios and steps for action to develop and implement better health literacy outcomes. By the end of the workshop, participants

– will have discussed the methodological approach for measuring navigational health literacy;

– will have heard of current data and developments regarding navigation health literacy in Austria, Germany and Switzerland;

– will have exchanged ideas and sustainable strategies to strengthen healthcare systems by developing navigational health literacy.

The four panelists will give short presentations on: HLS19, navigational health literacy research, the newly developed instrument measuring navigational health literacy in HLS19, key findings of their respective national health literacy surveys, and recommendations and current navigational health literacy initiatives in their countries. In a panel discussion, the panelists and workshop participants will discuss the presented findings and recommendations as well as the need for action regarding implementation strategies, hands-on initiatives, and sustainable solution approaches.

**Key messages:**

• Navigating healthcare systems and related information proves difficult for major parts of the general population.

• There is a need of health literate healthcare systems and organizations reducing the demands placed on patients by providing guidance and support.

**Speakers/Panellists:**

**Doris Schaeffer**

Bielefeld University, Bielefeld, Germany

**Lennert Griese**

Bielefeld University, Bielefeld, Germany

**Saskia De Gani**

Careum Center for Health Literacy, Zürich, Switzerland

**Robert Griebler**

Austrian National Public Health Institute, Vienna, Austria

